# The synergistic predictive value of hemoglobin glycation index and SYNTAX score for coronary artery disease complexity and long-term prognosis after percutaneous coronary intervention

**DOI:** 10.3389/fendo.2025.1727187

**Published:** 2026-01-13

**Authors:** Dongyue Jia, Maoling Jiang, Jie Feng, Junli Pan, Yuqi Tao, Sisi Wang, Si Liu, Hanxiong Liu, Zhen Zhang, Shiqiang Xiong, Lin Cai

**Affiliations:** 1 Department of Cardiology, Affiliated Hospital, Southwest Medical University, Luzhou, China; 2 Department of Cardiology, The Third People’s Hospital of Chengdu, Chengdu, China

**Keywords:** coronary artery disease, glucose metabolism, percutaneous coronary intervention, prediction model, prognosis

## Abstract

**Background:**

The complexity of coronary artery lesions and glucose metabolic disorders contributes to adverse long-term prognosis following percutaneous coronary intervention (PCI). This study aimed to evaluate the synergistic effect of coronary artery lesions—assessed by the SYNTAX score—and glucose metabolic disorders—quantified by the hemoglobin glycation index (HGI)—on predicting major adverse cardiac and cerebrovascular events (MACCEs) after PCI.

**Methods:**

A total of 609 coronary artery disease (CAD) patients undergoing PCI were enrolled in the final analysis. HGI was calculated by subtracting the predicted HbA1c (derived from fasting plasma glucose regression) from the observed HbA1c. Pearson’s coefficients were used for correlation analyses. Kaplan–Meier and Cox regression analyses were used to assess associations with MACCEs. Mediation analysis evaluated whether the SYNTAX score mediated the HGI–MACCEs relationship.

**Results:**

Patients with higher HGI and SYNTAX scores (≥1.16764 and >22, respectively) exhibited a significantly increased mortality risk (p = 0.0052) and more complex coronary lesions. Multivariable analysis confirmed HGI and SYNTAX score as independent predictors of MACCEs. Additionally, the SYNTAX score partially mediated the association between HGI and adverse outcomes, with a mediation proportion of 13.05%.

**Conclusion:**

The HGI and SYNTAX score exert a synergistic effect in predicting the severity of CAD and the risk of adverse prognosis after PCI. It highlights the necessity of integrating both metabolic and anatomical assessment indices for comprehensive risk stratification of CAD patients undergoing PCI.

## 1 Background

Although percutaneous coronary intervention (PCI) effectively improves outcomes in patients with coronary artery disease (CAD) by restoring coronary blood flow, approximately 20%–30% of these patients still experience recurrent cardiovascular events. Such recurrence remains a major contributor to ongoing morbidity and mortality in this population (1, 2). Therefore, identifying reliable predictors of adverse post-PCI outcomes is critical for optimizing the long-term management of CAD patients.

Diabetes mellitus is a well-established metabolic driver of CAD pathogenesis (3). While hyperglycemia, insulin resistance, and elevated HbA1c are known to correlate with CAD risk (4, 5), their clinical utility is limited by interindividual variability—variability influenced by non-glycemic factors such as erythrocyte lifespan and genetic polymorphisms (6). To address this limitation, the hemoglobin glycation index (HGI), the difference between observed HbA1c and predicted HbA1c (derived from fasting plasma glucose), was developed as a biomarker to quantify intrinsic glycation propensity (7). By capturing interindividual differences in hemoglobin glycation efficiency, HGI serves as a stable predictor of vascular complications, independent of a patient’s diabetes status (8).

Concurrently, the SYNTAX score is widely used to assess coronary anatomical complexity, and it has been shown to strongly predict long-term outcomes in patients undergoing PCI (9–11). However, despite integrating angiographic features of lesion severity, the SYNTAX score does not account for metabolic dysregulation—a key pathophysiological mechanism underlying CAD progression. Recent evidence has linked elevated HGI to poor post-PCI prognosis (12), but two critical questions remain unanswered: 1) Do HGI and the SYNTAX score exert a synergistic predictive effect on MACCEs in PCI patients? 2) Does coronary anatomical severity mediate the relationship between glucose metabolic disorders and clinical outcomes?

Although previous study have established associations between HGI and cardiovascular outcomes in ACS or diabetic populations (12), the synergistic effect of HGI and the SYNTAX score in predicting MACCEs after PCI remains underexplored. Importantly, no prior study has quantitatively evaluated whether coronary anatomical severity mediates the relationship between HGI and clinical outcomes. Our study addresses this gap by integrating metabolic and anatomical assessments in a prospective PCI cohort, thereby providing a novel risk-stratification framework that incorporates both intrinsic glycation propensity and lesion complexity.

## 2 Methods

### 2.1 Study population

A total of 609 consecutive hospitalized patients were enrolled from the Third People’s Hospital of Chengdu (Sichuan, China) between July 2018 and December 2022. Eligible patients underwent coronary angiography and received at least one stent implantation. The exclusion criteria were as follows: (1) a prior history of coronary artery bypass grafting; (2) critical structural heart disease requiring interventional treatment; (3) severe hepatic, respiratory, or renal insufficiency (with creatinine clearance <15 ml/min); (4) advanced hematological malignancies or solid tumors with a limited life expectancy; (5) in-hospital death; and (6) incomplete critical medical data (missing rate >10%). This study was approved by the Ethics Committee of the Third People’s Hospital of Chengdu and strictly adhered to the principles outlined in the Declaration of Helsinki. Written informed consent was obtained from all participants.

### 2.2 Data collection and definitions

Demographic characteristics, including age, sex, smoking status, history of diabetes and hypertension, and family history of CAD, were recorded. Smoking status was categorized as never, past, or current. Diabetes mellitus was indicated by a fasting plasma glucose level of ≥7.0 mmol/L, a two-hour postprandial glucose level of ≥11.1 mmol/L, or taking antidiabetic drugs (13). Hypertension was indicated by a systolic blood pressure of ≥140 mmHg and/or a diastolic blood pressure of ≥90 mmHg measured repeatedly at the clinic on different days, or taking antihypertensive drugs (14). Blood samples were collected from the participants before coronary angiography. The parameter values, including blood routine, fasting plasma glucose (FPG), blood lipid profile, HbA1c, and liver and kidney function, were measured by professionals in a standard basic laboratory with strict adherence to operational procedures.

HGI was calculated as follows: HGI = measured HbA_1c_ value - predicted HbA1c value (15). Simple linear regression estimation of the relationship between baseline FPG and baseline HbA1c in the 609 study participants yielded the following regression equation: HbA1c = 0.271 × FPG (mmol/L) + 4.979. Predicted HbA1c levels were then computed for each participant using their FPG values and this equation.

The baseline SYNTAX score was calculated using a web-accessible calculator (http://syntaxscore.com/). Preprocedural angiograms were analyzed by two independent interventional cardiologists blinded to clinical outcomes. The inter-observer reliability, assessed by the Intraclass Correlation Coefficient (ICC), was 0.89 (95% CI: 0.85–0.92), indicating excellent agreement. Any discrepancies were resolved through consensus after consultation with a third senior evaluator. All data were entered into a dedicated computer database and assessed for quality. All continuous variables were screened for outliers; non-physiological values were either excluded or Winsorized to ensure the statistical validity of the analyses.

### 2.3 Follow-up and endpoints

Follow-up assessments were conducted 1, 3, 6, and 12 months after discharge, and every 12 months thereafter through phone calls or in-person clinic visits. Clinical events that occurred during follow-up were documented by trained professionals. The primary endpoint was MACCEs, which included all-cause death, non-fatal myocardial infarction (MI), non-fatal stroke, and unplanned revascularization. The secondary endpoint included all-cause death, cardiac death, non-fatal MI, non-fatal stroke, and unplanned revascularization. Death from any cause was categorized as all-cause mortality. Unplanned revascularization was defined as ischemia-driven revascularization because of lesion progression or in-stent restenosis during follow-up after index procedures. MI and stroke were diagnosed based on internationally recognized guidelines.

### 2.4 Statistical analyses

Statistical analyses were performed using SPSS 24.0 and R version 4.2.1. Continuous and categorical variables were expressed as mean ± standard deviation and frequencies and percentages, respectively. Groups of continuous, normally distributed data were compared using ANOVA, while nonnormally distributed data were compared using the Kruskal–Wallis test. Categorical variables were compared using the Chi-squared test. Kaplan–Meier analysis was used to generate survival curves, and differences between groups were compared using the log-rank test. Variables for inclusion in the multivariable Cox regression model were selected based on a combination of statistical significance in univariate analysis (p < 0.10) and clinical relevance established in prior literature. The initial candidate variables from **Table 1** with a univariate p-value < 0.10 were age, HGI, diuretic use, insulin use, fibrinogen, bSS, BMI, and albumin. From these, age, HGI, fibrinogen, bSS, and BMI were retained in the final model. Albumin was excluded due to its collinearity with other nutritional/inflammatory markers, and diuretic/insulin use, while significant in univariate analysis, were no longer significant (p > 0.05) in the preliminary multivariate model and were thus omitted to preserve model parsimony. Clinically relevant variables like gender and diabetes history were considered for inclusion regardless of univariate p-value. For the Kaplan-Meier and Cox regression analyses, patients who were lost to follow-up or did not experience a MACCE by the end of the study period were right-censored at the time of their last follow-up assessment. Multivariable Cox proportional hazard regression was used to evaluate the predictive value of HGI for outcomes. Hazard ratios (HR) and 95% confidence intervals (CI) were calculated. A two-tailed p-value of <0.05 was considered statistically significant.

**Table 1:** Baseline characteristics.

Variable	Total (N = 609)	No Incident MACEs (N = 461)	Incident MACEs (N = 148)	p-value
Age, years	67.17 ± 10.97	66.32 ± 10.83	69.82 ± 11.03	0.001
Female, n (%)	165 (27.1%)	124 (26.9%)	41 (27.7%)	0.932
BMI (kg/m²)	24.40 ± 3.83	24.49 ± 3.84	24.14 ± 3.80	0.231
Current smoking, n (%)	217 (35.6%)	169 (36.7%)	48 (32.4%)	0.626
Hypertension, n (%)	444 (72.9%)	332 (72.0%)	112 (75.7%)	0.444
Diabetes mellitus, n (%)	438 (71.9%)	329 (71.4%)	109 (73.7%)	0.665
Prior stroke, n (%)	31 (5.1%)	25 (5.4%)	6 (4.1%)	0.657
Prior PCI, n (%)	65 (10.7%)	48 (10.4%)	17 (11.5%)	0.830
Atrial fibrillation, n (%)	18 (3.0%)	15 (3.3%)	3 (2.0%)	0.583
SBP (mmHg)	132.85 ± 21.56	133.10 ± 21.37	132.09 ± 22.17	0.764
FBG (mmol/L)	8.40 ± 4.10	8.45 ± 4.18	8.24 ± 3.86	0.571
HbA1c (%)	7.26 ± 1.78	7.18 ± 1.75	7.49 ± 1.85	0.078
LDL-C (mmol/L)	2.69 ± 0.91	2.71 ± 0.92	2.63 ± 0.86	0.842
HDL-C (mmol/L)	1.17 ± 0.83	1.18 ± 0.94	1.14 ± 0.31	0.368
Triglycerides (mmol/L)	1.54 ± 0.65	1.50 ± 0.64	1.65 ± 0.68	0.036
Fib (g/L)	3.83 ± 1.31	3.70 ± 1.26	4.23 ± 1.37	<0.001
hs-cTnT (pg/mL)	34.25 (12.28, 652.6)	30.41 (11.34, 602.8)	49.12 (13.82, 766.6)	0.038
Hcy (μmol/L)	17.20 ± 10.89	16.82 ± 10.15	18.37 ± 13.21	0.329
Alb (g/L)	39.29 ± 4.15	39.60 ± 4.10	38.32 ± 4.18	0.001
Serum creatinine (μmol/L)	92.59 ± 95.88	89.17 ± 72.86	103.23 ± 145.82	0.260
Uric acid (μmol/L)	379.12 ± 120.40	374.53 ± 117.10	393.42 ± 129.54	0.116
Total cholesterol (mmol/L)	4.37 ± 1.21	4.40 ± 1.21	4.26 ± 1.23	0.441
BNP (pg/mL)	102.8 (36.3, 313.1)	97.4 (32.9, 289.1)	117 (43.67, 417.03)	0.011cc
ACS presentation, n (%)	550 (90.3%)	413 (89.6%)	137 (92.6%)	0.755
Aspirin use, n (%)	590 (96.9%)	449 (97.4%)	141 (95.3%)	0.274
ACEI/ARB use, n (%)	299 (49.1%)	229 (49.7%)	70 (47.3%)	0.683
Insulin use, n (%)	126 (20.7%)	84 (18.2%)	42 (28.4%)	0.011
Diuretic use, n (%)	124 (20.4%)	83 (18.0%)	41 (27.7%)	0.015
Calcium channel blocker use, n(%)	171 (28.1%)	127 (27.6%)	44 (29.7%)	0.683
Oral hypoglycaemic agents, n (%)	311 (51.1%)	238 (51.6%)	73 (49.3%)	0.694
Lipid-lowering drug use, n (%)	597 (98.0%)	452 (98.1%)	145 (98.0%)	1.000
bSS	15.52 ± 8.64	14.72 ± 8.30	18.02 ± 9.22	<0.001
HGI	0.00 ± 1.39	−0.09 ± 1.35	0.28 ± 1.48	0.009

Data are presented as mean ± SD, median (IQR), or n (%). BMI, body mass index; SBP, systolic blood pressure; FBG, fasting blood glucose; HbA1c, glycated hemoglobin; LDL-C, low-density lipoprotein cholesterol; HDL-C, high-density lipoprotein cholesterol; Fib, fibrinogen; Hs-cTnT, high-sensitivity cardiac troponin T; Hcy, homocysteine; Alb, albumin; BNP, brain natriuretic peptide; ACS, acute coronary syndrome; ACEI/ARB, angiotensin-converting enzyme inhibitor/angiotensin receptor blocker; bSS, baseline SYNTAX score; HGI, hemoglobin glycation index; MACCEs, major adverse cardiac and cerebrovascular events.

## 3 Results

### 3.1 Baseline patient characteristics

**Figure 1:**
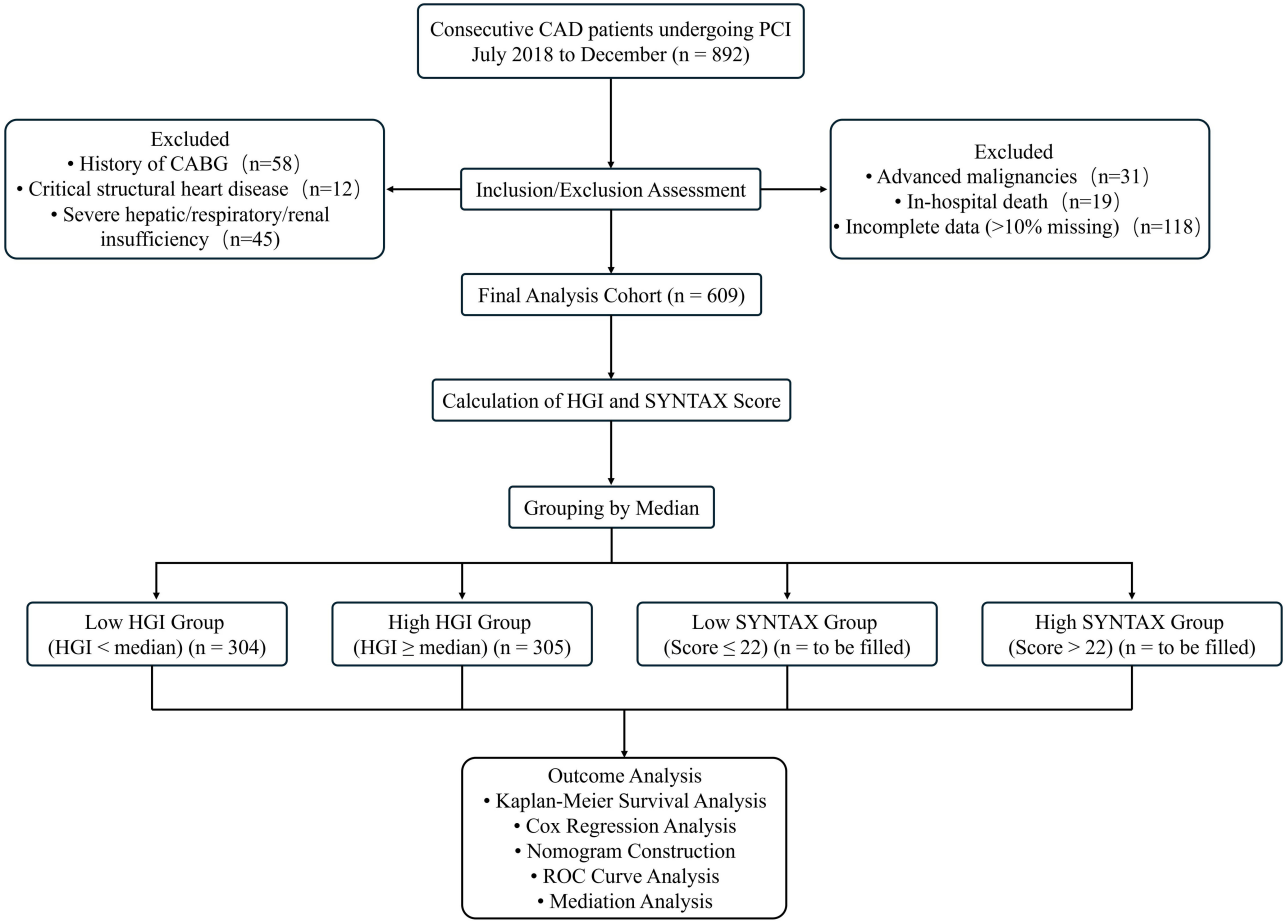
Study flow diagram for patient selection and analysis.

Baseline characteristics of the study population are presented in **Table 1**. Among the 609 participants (mean age 67.2 ± 11.0 years; median follow-up 30.4 months [IQR 24.3–34.3]), 148 (24.3%) experienced a MACCE during follow-up. Compared to the non-MACCEs group, patients who experienced a MACCE were significantly older and had a higher baseline SYNTAX score, fibrinogen level, and HGI value. They also had a greater prevalence of insulin and diuretic use, and lower BMI and serum albumin levels (all p < 0.05). However, sex distribution, smoking status, history of hypertension or diabetes, HbA1c, lipid profiles, and the use of aspirin or ACE inhibitors/ARBs did not differ significantly between the two groups (p ≥ 0.05). This study was approved by the Ethics Committee of the Third People’s Hospital of Chengdu and strictly adhered to the principles outlined in the Declaration of Helsinki. Written informed consent was obtained from all participants. The patient selection process is summarized in **Figure 1**.

### 3.2 Correlation between HGI and SYNTAX score

**Figure 2:**
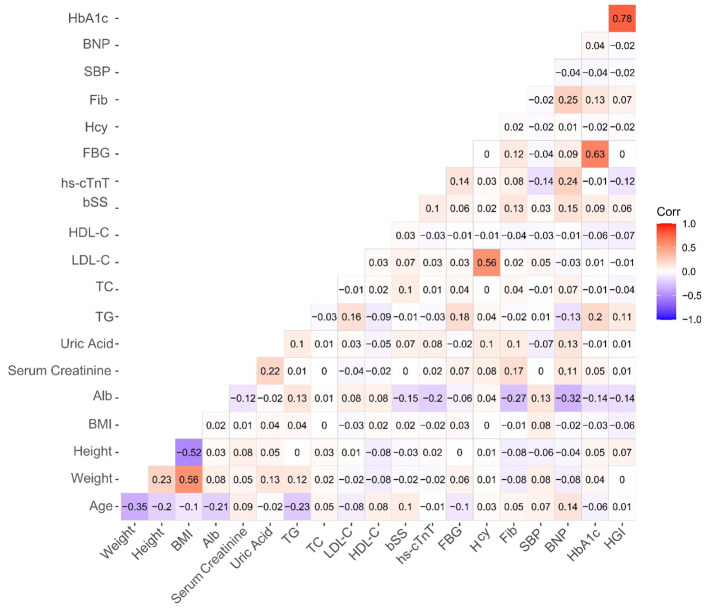
A heatmap of the correlation between different variables. Color intensity reflects correlation strength.

A heatmap visualization of correlations between different variables revealed a statistically significant but very weak positive association between HGI and the SYNTAX score (r = 0.06, p < 0.001, **Figure 2**).

### 3.3 Predictors of MACCE: univariate and multivariable Cox regression analyses

Univariate associations for potential predictors of MACCEs are presented in **Supplementary Table S1**. In the multivariable Cox regression analysis (**Table 2**), age (HR = 1.03, 95% CI: 1.01–1.04, p = 0.001), HGI (HR = 1.11, 95% CI: 0.98–1.25, p = 0.042), fibrinogen (HR = 1.19, 95% CI: 1.08–1.31, p < 0.001), baseline SYNTAX score (bSS) (HR = 1.03, 95% CI: 1.01–1.04, p = 0.002), and BMI (HR = 0.95, 95% CI: 0.90–1.00, p = 0.045) remained as independent predictors of MACCEs. The use of diuretics and insulin, which were significant in univariate analysis, were no longer significant in the multivariate model.

**Table 2:** Multivariate Cox regression analysis of the predictors of post-PCI MACCEs.

Variable	Univariate analysis		Multivariate analysis	
	HR 95% CI	p-value	95% CI (upper)	p-value
Age	1.03 (1.01–1.04)	0.001	1.03 (1.01–1.04)	0.001
HGI	1.18 (1.05–1.32)	0.004	1.11 (0.98–1.25)	0.042
Diuretics use	1.69 (1.17–2.42)	0.005	1.23 (0.84–1.79)	0.284
Insulin use	1.67 (1.16–2.39)	0.005	1.45 (0.98–2.13)	0.060
Fib	1.45 (0.98–2.13)	<0.001	1.19 (1.08–1.31)	<0.001
bSS	1.04 (1.02–1.06)	<0.001	1.03 (1.01–1.04)	0.002
BMI	0.95 (0.92-0.98)	0.004	0.95 (0.90 -1.00)	0.045

The predictive value of HGI and the SYNTAX score was further evaluated through a series of adjusted models (**Table 3**). In the unadjusted model, both HGI (HR = 1.18, 95% CI: 1.054–1.321, p = 0.004) and the preoperative SYNTAX score (HR = 1.039, 95% CI: 1.022–1.057, p < 0.001) were significantly associated with MACCEs. These associations persisted in Model I (adjusted for basic clinical variables) and Model III (the full model). In Model II (adjusted for laboratory parameters), the SYNTAX score remained a strong independent predictor (HR = 1.0307, 95% CI: 1.0132–1.0484, p < 0.001), while the association for HGI showed a trend towards significance (HR = 1.2247, 95% CI: 0.9943–1.5085, p = 0.057).

**Table 3:** Multivariable Cox regression for the risk of MACCEs post-PCI.

Model	HGI HR (95% CI)	p-value	Preoperative SYNTAX score HR (95% CI)	p-value
Unadjusted	1.18 (1.054, 1.321)	0.004	1.039 (1.022, 1.057)	<0.001
Model I	1.2159 (1.0693, 1.3826)	0.003	1.0349 (1.0044, 1.0402)	<0.001
Model II	1.2247 (0.9943, 1.5085)	0.057	1.0307 (1.0132, 1.0484)	<0.001
Model III	1.2494 (1.0214, 1.5283)	0.030	1.0297 (1.0119, 1.0478)	0.001

Model I (Basic Clinical Model): Adjusted for core demographic and clinical history variables: age, gender, BMI, hypertension, diabetes mellitus, smoking status, and previous PCI; Model II (Model I + Key Laboratory Parameters): Adjusted for all variables in Model I, plus serum creatinine, fibrinogen, and glycosylated hemoglobin (HbA1c). These laboratory values were added to account for renal function, inflammatory/coagulation status, and overall glycemic burden; Model III (Full Model): Adjusted for all variables in Model II, plus the use of lipid-lowering drugs (statins) and beta-blockers at discharge. These medications were added as they represent standard secondary prevention therapies that could influence long-term outcomes.

### 3.4 Risk stratification by HGI and SYNTAX score: Kaplan-Meier survival analysis

**Figure 3:**
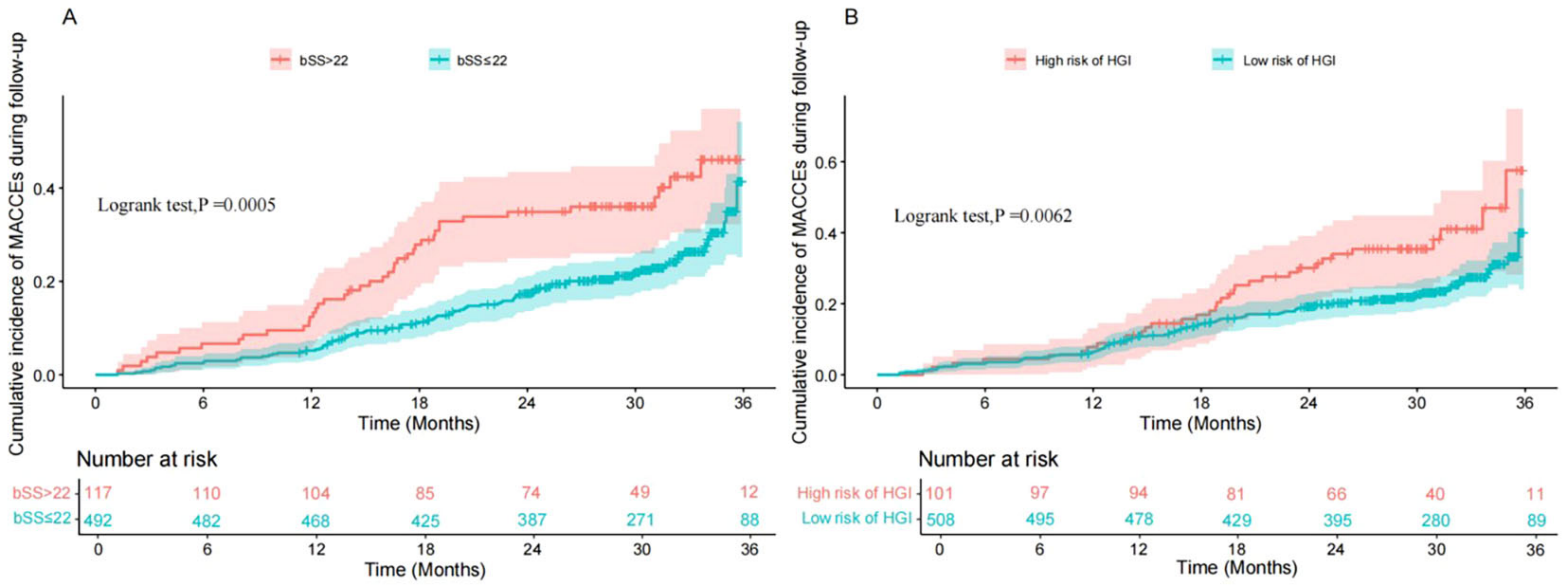
Kaplan–Meier curves for MACCE-free survival. A. Patients stratified by SYNTAX score: low-risk (≤ 22) vs intermediate/high-risk (> 22); log-rank P = 0.0005.B. Patients stratified by cohort-median hemoglobin glycation index (HGI): low-HGI (< 1.16764) vs high-HGI (≥ 1.16764); log-rank P = 0.0062.

Patients were stratified into groups based on the cohort median HGI (low-HGI: <1.16764; high-HGI: ≥1.16764) and a clinical SYNTAX score cutoff (low-risk: ≤22; intermediate/high-risk: >22). Kaplan-Meier survival analysis revealed that both an elevated SYNTAX score (**Figure 3A**; log-rank P = 0.0005) and a higher HGI (**Figure 3B**; log-rank P = 0.0062) were independently associated with a significantly increased risk of MACCEs during follow-up. This underscores the prognostic value of both coronary anatomic complexity and intrinsic glycemic variability for adverse clinical outcomes.

### 3.5 Predictive model performance: nomogram and ROC analysis

**Figure 4:**
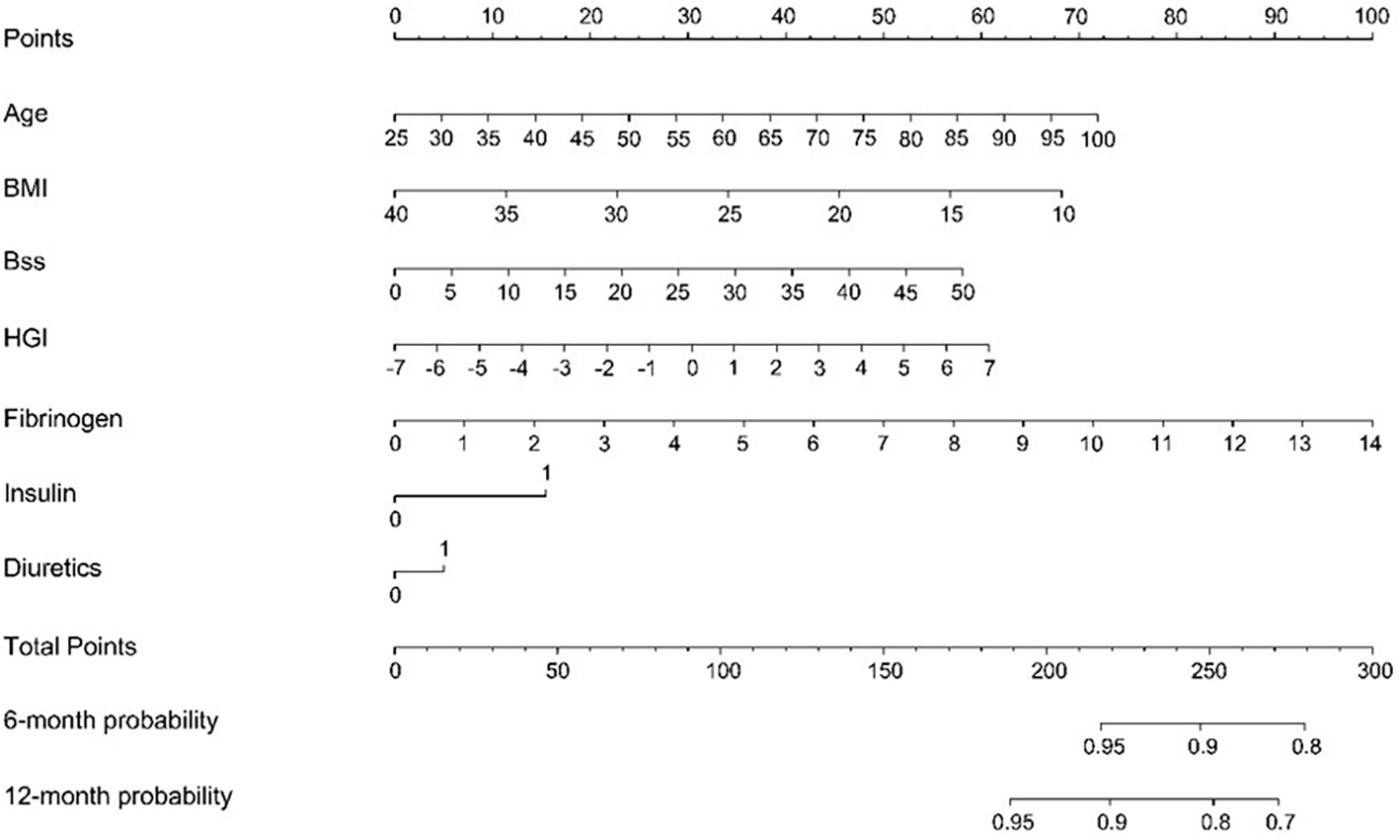
A nomogram predicting 6- and 12-month MACCE-free survival post-PCI. Assign points for each variable (upper scale), sum them to obtain the total points (top axis), and read the corresponding 6- or 12-month event-free probability on the bottom scale.

A seven-variable nomogram was constructed to estimate 6-month and 12-month MACCE-free survival probabilities after PCI (**Figure 4**). The nomogram incorporates individual patient data, converting them into an additive point score (range 0–160). Higher total points were consistently associated with lower event-free survival probabilities. Among the contributing variables, HGI and fibrinogen exhibited the steepest point gradients (approximately 7–8 points per 1-unit increase), identifying them as dominant drivers of risk within the model.

**Figure 5:**
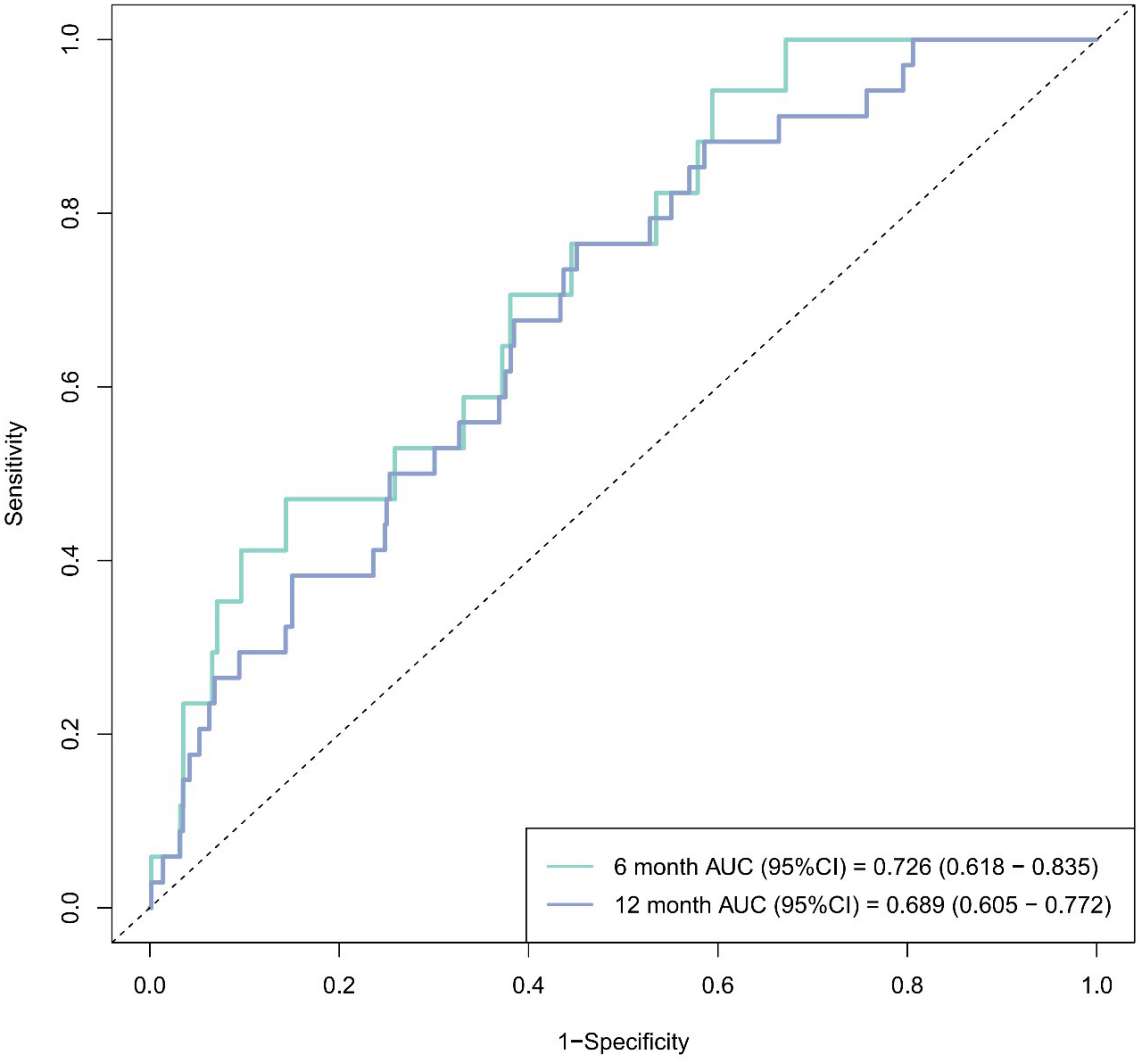
ROC curves for HGI’s MACCEs prediction at six and 12 months post-PCI. AUC: area under the curve, CI: confidence interval. Six- and 12-month AUCs = 0.726 (95% CI: 0.618–0.835) and 0.689 (95% CI: 0.605–0.772), respectively.

**Figure 6:**
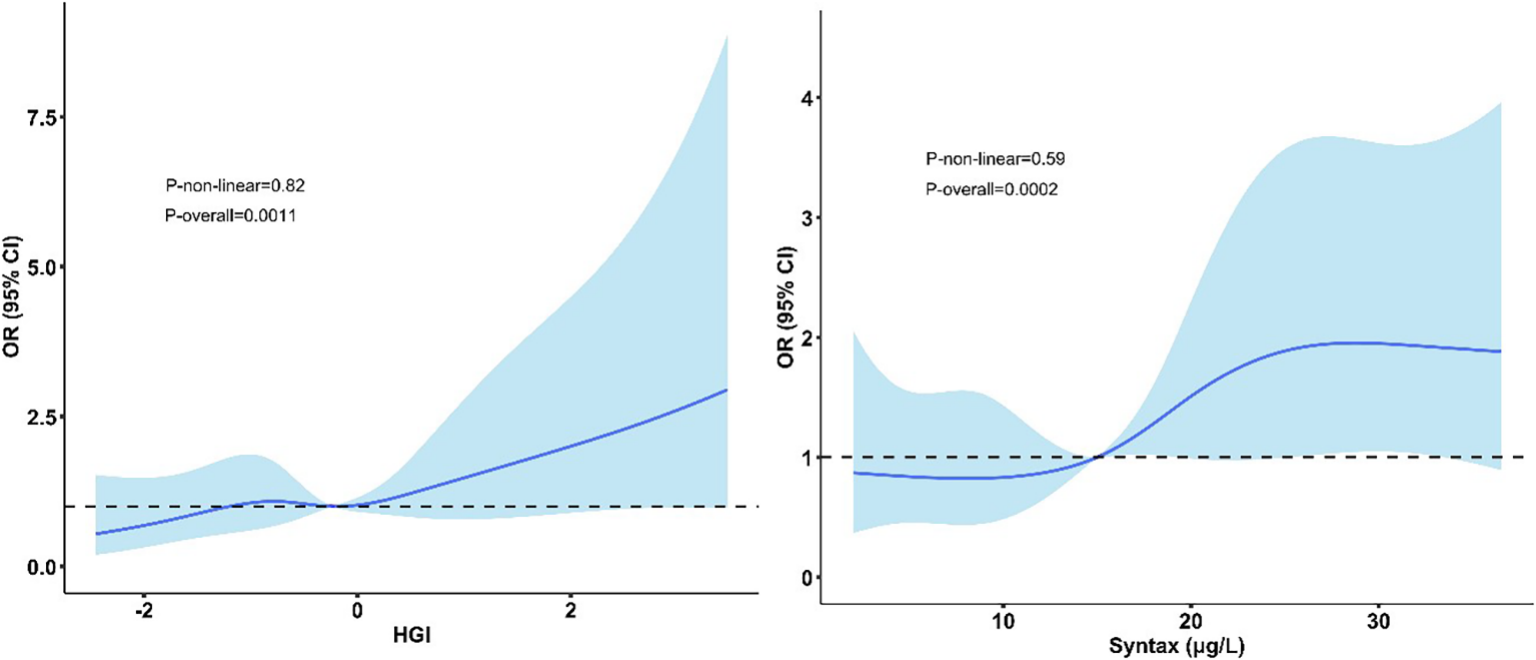
Restricted cubic spline curves for the association between HGI and SYNTAX score with MACCE risk. Solid lines represent hazard ratios (HRs). Shaded areas indicate 95% confidence intervals. P-overall and p-non-linear values are shown.

The predictive accuracy of HGI for MACCEs was further quantified using ROC curve analysis (**Figure 5**). The area under the ROC curve (AUC) for predicting 6-month MACCEs was 0.726 (95% CI: 0.618–0.835), indicating moderate discrimination. The AUC for 12-month MACCEs was 0.689 (95% CI: 0.605–0.772), indicating fair discrimination. The linear associations of HGI and SYNTAX score with MACCE risk are visually presented in **Figure 6**.

### 3.6 Mediation analysis

**Figure 7:**
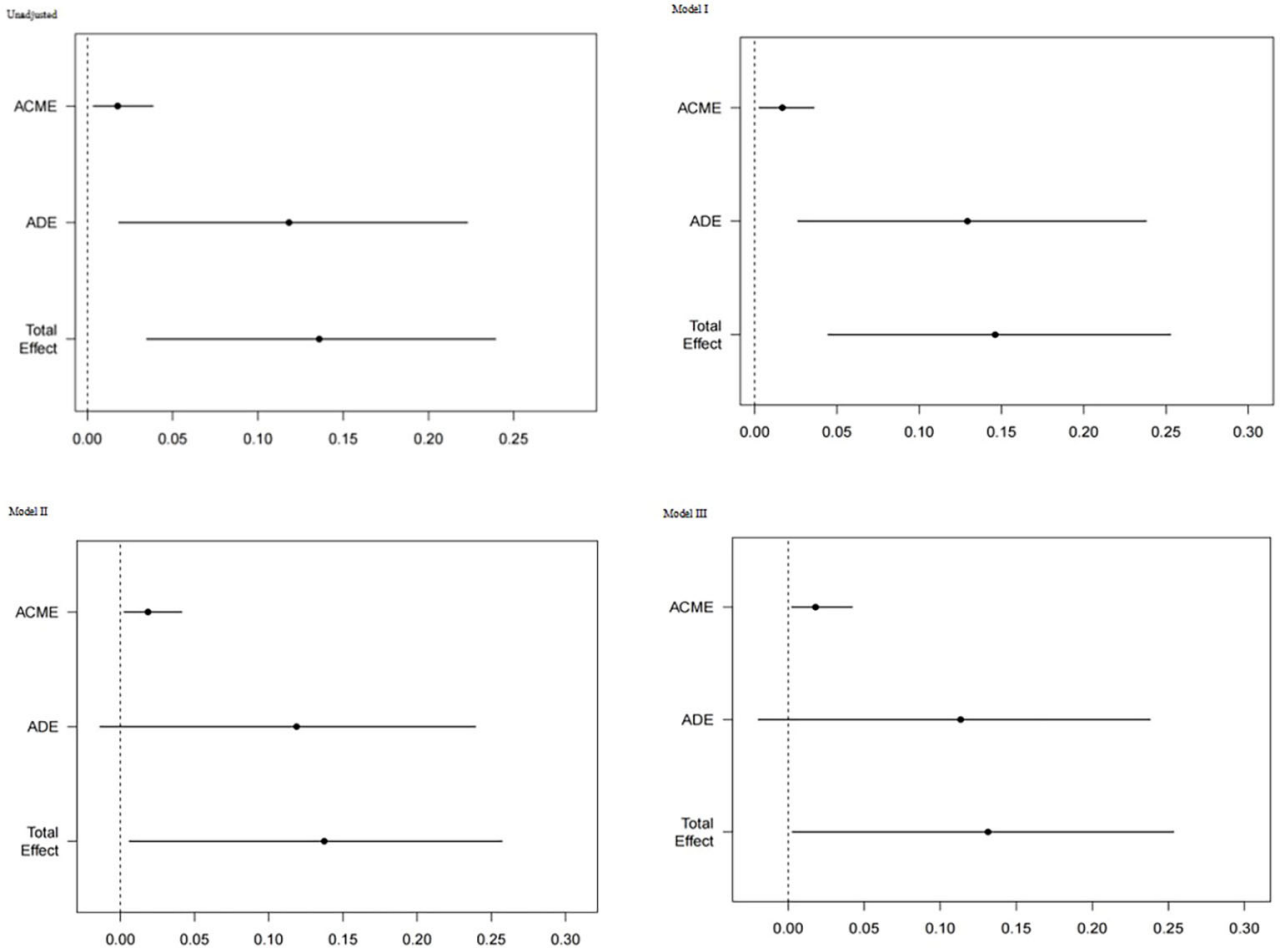
The results of mediation analysis. A mediation pathway diagram of the relationship between HGI and MACCEs via SYNTAX score. The indirect effect (β = 0.018, 95% CI: 0.000–0.036) and proportion mediated (13.05%, 95% CI: 1.90–21.95) were derived from 1,000 bootstrap resamples. The p-values for the indirect and direct effects were 0.006 and 0.020, respectively.

To explore the potential pathway linking HGI to adverse outcomes, a mediation analysis was performed with the SYNTAX score as the mediator. In all models, the total effect of HGI on MACCE risk was significant (e.g., unadjusted model: HR = 1.146, 95% CI: 1.034–1.269, p = 0.014). The analysis revealed a significant indirect effect, indicating that HGI elevates MACCE risk partly through its association with a higher SYNTAX score (unadjusted indirect HR = 1.018, 95% CI: 1.000–1.036, p = 0.006). The direct effect of HGI, independent of the SYNTAX score, was also significant (unadjusted direct HR = 1.125, 95% CI: 1.016–1.247, p = 0.020). Consequently, the SYNTAX score mediated 13.05% (95% CI: 1.90–21.95, p = 0.020) of HGI’s total effect on MACCEs in the unadjusted model. This significant mediation effect persisted across all adjusted models (**Table 4**, **Figure 7**).

**Table 4:** Mediation analysis of the relationship between HGI and MACCEs via SYNTAX score.

Model	Total effect HR (95% CI)	p-value	Indirect effect HR (95% CI)	p-value	Direct effect HR (95% CI)	p-value	PM (%) (95% CI)	p-value
Unadjusted	1.146 (1.034–1.269)	0.014	1.018 (1.000–1.036)	0.006	1.125 (1.016–1.247)	0.020	13.05 (1.90–21.95)	0.020
Model I	1.157 (1.043–1.284)	0.004	1.017 (1.000–1.034)	0.020	1.138 (1.024–1.265)	0.012	11.50 (2.53–22.89)	0.024
Model II	1.147 (1.012–1.301)	0.046	1.019 (0.999–1.039)	0.014	1.126 (0.992–1.278)	0.041	13.56 (−0.40–24.71)	0.037
Model III	1.140 (1.006–1.293)	0.044	1.018 (0.998–1.039)	0.016	1.120 (0.985–1.274)	0.037	13.70 (−1.13–23.94)	0.045

Data are derived from 1,000 bootstrap resamples. Indirect effect and proportion mediated (PM) are reported with 95% confidence intervals. HR: hazard ratio, CI: confidence interval. Model I (Basic Clinical Model): Adjusted for core demographic and clinical history variables: age, gender, BMI, hypertension, diabetes mellitus, smoking status, and previous PCI. Model II (Model I + Key Laboratory Parameters): Adjusted for all variables in Model I, plus serum creatinine, fibrinogen, and glycosylated hemoglobin (HbA1c). These laboratory values were added to account for renal function, inflammatory/coagulation status, and overall glycemic burden;Model III (Full Model): Adjusted for all variables in Model II, plus the use of lipid-lowering drugs (statins) and beta-blockers at discharge. These medications were added as they represent standard secondary prevention therapies that could influence long-term outcomes.

## 4 Discussion

This study systematically assessed the synergistic prognostic value of the hemoglobin glycation index (HGI) and SYNTAX score in patients undergoing percutaneous coronary intervention (PCI) by integrating metabolic and anatomical indicators. For the first time, we quantified the mediating role of coronary lesion severity in the relationship between HGI and clinical outcomes. Key findings included: (1) a significant synergistic effect of HGI and SYNTAX score in predicting major adverse cardiac and cerebrovascular events (MACCEs); (2) the SYNTAX score mediated 13.05% of the total effect of HGI on MACCEs; and (3) these synergistic and mediating effects remained robust after multivariate adjustment.

Our analysis demonstrated that patients with both elevated HGI (≥1.16764) and high SYNTAX score (>22) had a significantly higher risk of MACCEs compared to those with only one risk factor (Log-rank P = 0.0052). This synergy suggests that metabolic disturbances and coronary anatomical complexity may interact to promote cardiovascular events. Pathophysiologically, the inherent glycation propensity associated with high HGI could accelerate the progression of atherosclerosis and increase plaque instability by fostering the accumulation of advanced glycation end products (AGEs), aggravating oxidative stress, and impairing endothelial function (16, 17). Concurrently, complex coronary lesions, indicated by a high SYNTAX score, reflect a greater burden of vascular disease. When coexisting, these factors may exacerbate each other’s adverse effects on prognosis through a ‘metabolic-anatomical-functional’ vicious cycle, resulting in a multiplicative increase in risk.

Mediation analysis revealed that SYNTAX score played a partial mediating role in the relationship between HGI and MACCEs, with a mediation proportion of 13.05% (95% CI: 1.90-21.95). This finding has important mechanistic significance, indicating that part of the adverse effect of high HGI on prognosis is achieved by aggravating the complexity of coronary artery lesions. Specifically, long-term exposure to a high glycation environment may promote the progression of coronary atherosclerotic plaques, increase lesion complexity (such as bifurcation lesions, calcified lesions, etc.), thus manifesting as a higher SYNTAX score, and ultimately leading to an increased risk of adverse events. However, it is worth emphasizing that the vast majority (about 87%) of the HGI effect was not mediated by SYNTAX score, suggesting that HGI may affect prognosis through other non-anatomical pathways, such as microvascular dysfunction, systemic inflammation activation, increased platelet reactivity, and direct myocardial toxicity, etc. (17–21). Therefore, in clinical assessment, relying solely on anatomical indicators may not be sufficient to fully capture all risk dimensions of high HGI patients.

Compared with traditional HbA1c, HGI showed better prognostic stratification ability in this study. Multivariate Cox regression showed that HGI was an independent predictor of MACCEs (HR = 1.18, p = 0.004), while HbA1c did not reach statistical significance (HR = 1.08, p = 0.077). HGI more accurately reflects the degree of intrinsic metabolic disorder by correcting for the effects of non-glycemic factors such as inter-individual differences in red blood cell lifespan and genetic variations, which may be the reason for its superior predictive value over HbA1c (7, 8). Baseline HbA1c levels showed no significant difference in the MACCEs vs non-MACCEs groups (7.49% vs. 7.18%, p = 0.078), whereas baseline HGI was markedly elevated in the MACCEs group (0.28 vs. -0.09, p = 0.009). This divergence underscores critical pathophysiological and clinical distinctions between these two glycemic metrics. Specifically, HGI quantifies intrinsic hemoglobin glycation propensity by comparing observed HbA1c with predicted values based on fasting glucose. This adjustment isolates metabolic dysregulation from non-glycemic confounding factors, which explains its stronger association with vascular injury (22–25). An observational cohort study in China reported a U-shaped relationship between HGI levels and the incidence of MACCEs in acute coronary syndrome patients with or without diabetes. Lower and higher HGI could increase the risk of poor outcomes (26). Another extensive cohort study confirmed a U-shaped relationship between HGI and MACE occurrence within three years in patients with diabetes and established CAD. Low and high HGI were linked to a greater MACE risk at the three-year follow-up. Additionally, patients with low HGI had a higher chance of CV death. These findings imply that HGI can help assess this population’s risk and prognosis. In previous ACS studies, HbA1c exhibited non-linear (U-shaped) relationships with outcomes (12), which complicated clinical interpretation. Conversely, we observed a consistent linear risk gradient for HGI (p-non-linear = 0.82), enhancing its utility for risk stratification. This stability reflects HGI’s characterization of inherent metabolic phenotypes resistant to short-term glycemic fluctuation (8). At the six-month follow-up, HGI demonstrated superior predictive accuracy (area under the curve = 0.726) when compared with HbA1c. This advantage persisted after multivariable adjustment, confirming HGI’s unique value in identifying high-risk PCI patients missed by conventional metrics.

This study elucidated the SYNTAX score’s mediating function in the correlation between the HGI and adverse cardiovascular outcomes, including MACCEs and unplanned revascularization following PCI, thereby amalgamating previous evidence into a holistic pathway to inform clinical decision-making. Globally, the number of patients receiving coronary intervention has risen consistently. Individuals with coronary heart disease, particularly those with type 2 diabetes mellitus or intricate coronary artery lesions, have a high susceptibility to recurring adverse cardiovascular incidents (10, 27). Therefore, the prompt recognition of high-risk patients with unfavorable prognoses is imperative. In clinical practice, HGI evaluation in patients with CAD enhances overall disease assessment and facilitates the development of more effective, personalized treatment and management strategies. Moreover, understanding the correlation between HGI and CAD, and cardiovascular adverse event severity enables physicians to better evaluate patient risk and promptly identify and address potential complications. Unlike earlier cohorts restricted to ACS or diabetes, our study enrolled a broader CAD population undergoing PCI, followed them for a longer interval, and applied rigorous mediation analysis. The observed synergy between HGI and the SYNTAX score, together with the 13.05% partial mediation effect, underscores the necessity of combining metabolic and anatomical indices for comprehensive prognostic evaluation. The mediation effect remained consistent and statistically significant across all models; however, the wider confidence intervals in the fully adjusted analyses revealed statistical imprecision and a modest effect size. This underscores that while coronary anatomical complexity is a measurable and significant pathway, it represents only one part of the mechanistic link between aberrant glycation and clinical outcomes.

Although the SYNTAX score mediated only 13.05% of the total effect of HGI on MACCEs, this finding underscores a measurable pathway through which metabolic dysregulation influences outcomes via coronary anatomical complexity. The majority of HGI’s effect, however, remained unexplained by lesion severity, suggesting additional mechanisms—such as systemic inflammation, endothelial dysfunction, plaque vulnerability, and microvascular impairment—may contribute to the elevated risk observed in high-HGI patients (16–19). These non-anatomical pathways highlight the multifactorial nature of glycemic-related vascular injury and support the integration of both metabolic and anatomical assessments for comprehensive risk stratification.

This study has limitations. First, HGI was derived from fasting plasma glucose rather than mean blood glucose levels. While clinically practical, this method may inadequately capture postprandial glycemic contributions, particularly in diabetes, potentially leading to underestimated predicted HbA1c and overestimated HGI. Such non-differential misclassification would attenuate the true HGI-MACCEs association. Nonetheless, the significant association observed confirms HGI’s prognostic robustness. Further validation using continuous glucose monitoring remains warranted. Second, the study’s single-center retrospective cohort design limits our ability to establish a causal link between HGI, SYNTAX score, and adverse cardiovascular events following PCI. Additionally, despite adjusting for known cardiac risk factors, the study’s observational design means that all confounding variables might not have been accounted for, leaving room for potential residual confounding effects. Third, selection bias was possible because we could only simultaneously select participants with HbA1c and FPG values to calculate HGI. Therefore, our results need validation using a multicenter prospective study. Fourth, while we employed bootstrap resampling to enhance the reliability of our mediation analysis, the modest sample size (n=609) may limit the stability of the mediation effect estimate (13.05%, 95% CI: 1.90-21.95). The relatively wide confidence intervals indicate some uncertainty in the precise magnitude of this effect, and validation in larger cohorts is warranted to establish more precise estimates of the mediation proportion.

## 5 Conclusion

This study confirmed that HGI and SYNTAX score are significantly associated with an increased risk of adverse outcomes after PCI. The SYNTAX score partially mediates the relationship between HGI and adverse cardiovascular outcomes, including MACCEs and unplanned revascularization. Integrating the HGI and SYNTAX score enriches the evidence for HGI as a risk factor in CVD, and it may facilitate a more precise clinical management of patients with CVD.

## Data Availability

The raw data supporting the conclusions of this article will be made available by the authors, without undue reservation.
