# Supplementary material for: The synergistic predictive value of hemoglobin glycation index and SYNTAX score for coronary artery disease complexity and long-term prognosis after percutaneous coronary intervention
Source: Front Endocrinol (Lausanne). 2026 Jan 13;16:1727187. doi: 10.3389/fendo.2025.1727187 (PMC12834722; doi:10.3389/fendo.2025.1727187)
Supplement: Supplementary file 1 [file Table1.docx]

**Table S1.Univariate Cox regression analysis of MACCE.**

| **Variable** | **HR** | **95% CI (Lower)** | **95% CI (Upper)** | ***p*-value** |
| --- | --- | --- | --- | --- |
| **Sex** | 1.12 | 0.78 | 1.61 | 0.538 |
| **Age** | 1.03 | 1.01 | 1.04 | <0.001 |
| **BMI** | 0.95 | 0.92 | 0.98 | 0.004 |
| **Occasional smoker** | 1.03 | 0.66 | 1.59 | 0.911 |
| **Regular smoker** | 0.84 | 0.58 | 1.21 | 0.355 |
| **History of hypertension** | 1.28 | 0.88 | 1.87 | 0.193 |
| **Diabetes mellitus** | 1.08 | 0.75 | 1.56 | 0.682 |
| **Alb** | 0.94 | 0.90 | 0.98 | 0.002 |
| **Serum creatinine** | 1.00 | 1.00 | 1.00 | 0.177 |
| **Uric acid** | 1.00 | 1.00 | 1.00 | 0.032 |
| **TG** | 0.95 | 0.85 | 1.06 | 0.343 |
| **TC** | 1.00 | 0.99 | 1.01 | 0.793 |
| **LDL-C** | 1.00 | 0.92 | 1.08 | 0.988 |
| **HDL-C** | 0.87 | 0.68 | 1.11 | 0.263 |
| **CCB use** | 1.11 | 0.78 | 1.58 | 0.569 |
| **ACEI/ARB use** | 0.94 | 0.68 | 1.30 | 0.715 |
| **bSS** | 1.04 | 1.02 | 1.06 | <0.001 |
| **PCI history** | 0.91 | 0.54 | 1.54 | 0.723 |
| **History of atrial fibrillation** | 0.71 | 0.23 | 2.24 | 0.564 |
| **HGI** | 1.18 | 1.05 | 1.32 | 0.004 |
| **hs-cTnT** | 1.00 | 1.00 | 1.00 | 0.985 |
| **FBG** | 0.99 | 0.95 | 1.03 | 0.542 |
| **Homocysteine** | 1.00 | 1.00 | 1.00 | 0.571 |
| **Fib** | 1.22 | 1.11 | 1.33 | <0.001 |
| **Aspirin use** | 0.71 | 0.33 | 1.56 | 0.399 |
| **Insulin use** | 1.67 | 1.16 | 2.39 | 0.005 |
| **Hypoglycemic agents** | 0.89 | 0.64 | 1.23 | 0.480 |
| **Lipid-lowering agent use** | 0.97 | 0.31 | 3.06 | 0.963 |
| **History of stroke** | 0.85 | 0.38 | 1.93 | 0.704 |
| **SBP** | 1.00 | 0.99 | 1.01 | 0.591 |
| **BNP** | 1.00 | 1.00 | 1.00 | 0.097 |
| **Diuretics use** | 1.69 | 1.17 | 2.42 | 0.005 |
| **UA** | 1.63 | 0.86 | 3.10 | 0.136 |
| **NSTEMI** | 1.70 | 0.85 | 3.39 | 0.135 |
| **STEMI** | 1.49 | 0.76 | 2.92 | 0.240 |
| **HbA1c** | 1.08 | 0.99 | 1.18 | 0.077 |

HR, hazard ratio; CI, confidence interval; MACCE, major adverse cardiovascular and cerebrovascular events. *P < 0.05 was considered statistically significant.
